# Monitoring the stress physiology of free-ranging mugger crocodiles (*Crocodylus palustris*) across diverse habitats within Central Gujarat, India

**DOI:** 10.1093/conphys/coae035

**Published:** 2024-06-05

**Authors:** Brinky Desai, Tathagata Bhowmik, Rohith Srinivasan, Nikhil Whitaker, Ratna Ghosal

**Affiliations:** Biological and Life Sciences, School of Arts and Sciences, Ahmedabad University, Commerce Six Roads, Navrangpura, Ahmedabad 380009, Gujarat, India; Biological and Life Sciences, School of Arts and Sciences, Ahmedabad University, Commerce Six Roads, Navrangpura, Ahmedabad 380009, Gujarat, India; Biological and Life Sciences, School of Arts and Sciences, Ahmedabad University, Commerce Six Roads, Navrangpura, Ahmedabad 380009, Gujarat, India; Madras Crocodile Bank Trust, Post Bag No 4, Mahabalipuram, Chennai 603104, Tamil Nadu, India; Biological and Life Sciences, School of Arts and Sciences, Ahmedabad University, Commerce Six Roads, Navrangpura, Ahmedabad 380009, Gujarat, India

**Keywords:** Conflict, faecal glucocorticoid metabolite, muggers, rural, urban**Abbreviations:** CV, coefficients of variation; EIA, enzyme immunoassay; fGCM, faecal glucocorticoid metabolite; GC, glucocorticoid; GCM, glucocorticoid metabolite; HMC, human–mugger conflict; LME, linear mixed effect; OD, optical density

## Abstract

Animals face several challenges in their natural environment, and to cope with such conditions, they may exhibit contrasting physiological responses that directly affect their overall well-being and survival. In this study, we assessed physiological responses via faecal glucocorticoid metabolite (fGCM) measurements in free-ranging mugger crocodiles inhabiting diverse habitats in Gujarat, India. We sampled muggers within Charotar, a rural area (Zone A) with local people having high tolerance towards the presence of muggers, and Vadodara, a region having both urban (Zone B) and rural (Zone C) areas with high levels of human–mugger conflict (HMC). Further, muggers in Vadodara live in water bodies that are mostly polluted due to sewage disposal from adjoining chemical industries. To measure fGCM (mean ± SEM, ng/g dry faeces) levels in muggers, scats were collected during both breeding (*N* = 107 scats) and non-breeding (*N* = 22 scats) seasons from all three zones. We used captive muggers (a focal enclosure) to biologically validate (via capture and restraint) the selected fGCM assay (11-oxoetiocholanolone assay). We showed a significant (*P* < 0.05) 11-fold increase in fGCM levels between pre-capture (540.9 ± 149.2, *N* = 11) and post-capture (6259.7 ± 1150.5, *N* = 11) samples. The validated assay was applied to free-ranging muggers during the breeding season, and Zone A showed significantly (*P* < 0.05) lower fGCM levels (542.03 ± 71.3) compared to muggers of Zone B (1699.9 ± 180.8) and Zone C (1806.4 ± 243.2), both zones having high levels of HMC with polluted water bodies. A similar contrast in fGCM levels was also observed during the non-breeding season. Overall, the study demonstrated that fGCM levels in muggers varied across habitats, and such variation could be due to a multitude of ecological factors that the species experience in their immediate local environment. Moreover, high fGCM levels in muggers of Vadodara during both breeding and non-breeding seasons may indicate a condition of chronic stress, which could be maladaptive for the species.

## Teaser

The study demonstrated contrasting physiological responses via faecal glucocorticoid metabolite (fGCM) measurements in free-ranging muggers across diverse habitats within the western part of India. Significant differences in fGCM levels across populations may be due to a combination of ecological and anthropogenic factors that the muggers experience in their immediate local environment.

## Introduction

In today’s world, with a high rate of globalization and exponential growth in the human population, most wildlife species, including crocodiles, face diverse sets of threats endangering their survival in the native environment. Crocodilians are large reptilian species and are globally distributed in both freshwater and marine water habitats. According to the International Union for Conservation of Nature (IUCN) Red List, there are 23 extant species of crocodiles, of which 7 are ‘critically endangered’, 4 are ‘vulnerable’ and 12 are of ‘least concern’ (Choudhury and De Silva, 2013; Vashistha *et al.*, 2020). Some significant threats that most crocodile species face include habitat degradation, pollution, poaching and trafficking (Somaweera *et al.*, 2019). Several crocodilian species also experience a high degree of conflict with adjoining human settlements, particularly with regard to getting access to water bodies (Fukuda *et al.*, 2014; Corvera *et al.*, 2017; García-Grajales and Buenrostro-Silva, 2019; Khan *et al.*, 2020). For example, a study on saltwater crocodiles (*Crocodylus porosus*) reported 665 attacks on humans across the entire Indonesian archipelago between 2010 and 2019, of which 47% were lethal and 53% were non-lethal (Henkanaththegedara *et al.*, 2023). The frequency and severity of the attacks were positively correlated with human activities, as local people depend upon the adjoining water bodies for daily chores, including bathing, defecation, fishing, swimming and commuting on boats, thus coming into close contact with the large reptiles. Since crocodiles are ectothermic animals, they also need open basking lands to regulate body temperature (Grigg and Gans, 1993), which places them in direct conflict with human activities on lands, as well (Vyas and Vasava, 2019; Oommen, 2021). Moreover, conflict incidents usually reach a peak during the breeding season, as crocodiles are ‘hole-nesters’, having on-shore dens with extensive guarding, and parents actively defend the nests and the hatchlings (Henkanaththegedara *et al.*, 2023). Globally, such conflict situation between humans and crocodiles is quite common (Wallace *et al.*, 2011; Fukuda *et al.*, 2014; Brien *et al.*, 2017; Brackhane *et al.*, 2018; Das and Jana, 2018; Uluwaduge *et al.*, 2018; García-Grajales and Buenrostro-Silva, 2019; Khan *et al.*, 2020). However, a contrasting situation exists in a few crocodile habitats, where local people peacefully coexist with significant populations of large reptiles (Van Der Ploeg *et al.*, 2011). Such high tolerance of the locals towards the crocodiles is primarily because of the representation and importance of the species in traditional folklore and due to the religious beliefs of the locals considering crocodiles as vehicles or companions of ‘Gods’ (Pooley, 2016; Cavalier *et al.*, 2022).

Apart from experiencing contrasting attitudes of the locals, crocodiles also face several other challenges in their natural environments. For instance, habitat fragmentation caused by infrastructure development, like the building of railway tracks, highways and electric lines, has adversely affected the movement of several crocodilian species (Aresco, 2005; Pethiyagoda *et al.*, 2015; Vyas and Vasava, 2019), resulting in fatalities due to collision and electrocution. Further, the Nile crocodile (*Crocodylus niloticus*) populations in Zimbabwe faced a severe threat due to habitat deterioration, as most freshwater habitats were taken up for agricultural purposes and were polluted due to industrial discharge. The species also suffered a high harvest rate due to the meat and skin trade. Further, Nile crocodiles also faced intense conflict with humans, resulting in multiple fatalities, and this prompted people to kill crocodiles out of rage, further threatening the population status of the species (Utete, 2021). Water pollution has also significantly impacted several crocodilian species by deteriorating the quality of habitats (Maphanga *et al.*, 2022; Thirion *et al.*, 2022). For example, the accumulation of pesticides and organophosphates (used to combat malaria) in Lake St Lucia, South Africa, impacted the health of Nile crocodiles (Humphries *et al.*, 2021), and the discharge of heavy metals like mercury in high concentration in several water systems of southeastern Mexico had a significant impact on the well-being of the Morlet’s crocodiles (*Crocodylus moreletii*) (Buenfil-Rojas *et al.*, 2022). Studies on species belonging to the Alligatoridae (alligator and caiman) family (Guillette Jr *et al.*, 1996; Tavalieri *et al.*, 2021) have demonstrated that pollutants act as endocrine disruptors, impacting the natural reproductive function of the organisms. However, to our knowledge, no systematic study has been conducted on species belonging to the Crocodylidae family to understand how these pollutants may impact the physiology of the apex predators, which warrants further investigations.

**Figure 1:**
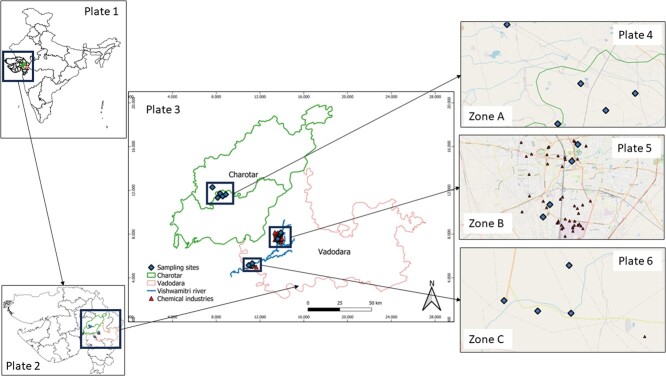
Map representing study sites in central Gujarat, India, was created using Quantum Geographic Information System (Version 3.28.1) mapping software and Google map plugins: Plate 1 represents India, showing the state of Gujarat, which is highlighted; Plate 2 represents Charotar (with green border) and Vadodara (with red border) regions within the state of Gujarat; Plate 3 represents zones A, B and C within the Charotar and Vadodara regions, the sampling sites within the three zones and the locations of chemical industries within a 5 km radius of each of the sampling sites; Plates 4, 5 and 6 represent closer view (Zoom: 16×) of Zones A, B and C, respectively, through the open street map plugin in Q-GIS. The map shows no chemical industry in Zone A, and 49 chemical industries are located in Zone B and 1 in Zone C.

Mugger crocodile is a freshwater crocodilian species that lives in diverse habitats, ranging from semi-arid deserts to ponds and lakes in villages/rural areas to irrigation canals and sewage drains in urban cities (Desai *et al.*, 2022), and it is spread in ~50 small pockets within India and the subcontinent (Mobaraki *et al.*, 2021). Muggers are ‘vulnerable’ according to the IUCN Red List and are provided legal protection under Schedule I of the Wildlife Protection Act of India (1972). On a global scale, mugger crocodiles rank third in the human–crocodile conflict, following the saltwater and Nile crocodiles (Sideleau and Britton, 2013). India saw a fivefold increase in human–mugger conflict (HMC) incidences over the last two decades, from 57 incidents in 2001–2010 to 338 reported incidents in 2011–2020 (Mobaraki *et al.*, 2021). One such HMC hotspot within India is the Vadodara region, which has a large population of muggers, particularly in the Vishwamitri River (Vyas, 2018; Bhowmik and Bhatt, 2023). Vadodara is a mix of urban and rural zones (panchayat.gujarat.gov.in), supporting a large number of migrant workers (Desai, 2017), and is characterized by the presence of several chemical industries that lie mostly within the urban areas (Magadum *et al.*, 2017; Bhangaonkar and Patel, 2019) (Fig. 1). Most rivers within Vadodara support a significant population of muggers (Magadum *et al.*, 2017; Vyas, 2018; Bhangaonkar and Patel, 2019; Pagdand, 2019; Meena *et al*., 2023; Vyas, 2023), but these riverine habitats within the urban zones greatly suffer from sewage pollution from neighbouring industries (Kumar and Goel, 2014; Swain *et al.*, 2022; Yadav and Yadav, 2023). Further, most of the locals in the rural areas of Vadodara heavily depend on the rivers for daily chores, including the collection of potable water (Vyas, 2013).

Contrastingly, 45 km away from Vadodara is the Charotar region (including both Kheda and Anand districts, Makwana, 2017; Patel *et al.*, 2020), which is primarily a rural area (panchayat.gujarat.gov.in) with a large proportion of agricultural lands (Voluntary Nature Conservancy, 2022) and no industries (Fig. 1). The Charotar locals, for generations, have maintained a coexistence with free-ranging muggers and preserved the cultural significance of the mugger crocodile as the sacred vehicle, ‘Vahan/vahana’, of ‘Goddess Khodiyar’ (Voluntary Nature Conservancy, 2017, 2018, 2019, 2020, 2021, 2022; Pooley, 2022). As per the survey conducted by the Voluntary Nature Conservancy in 2021, the mugger crocodile population in the Charotar region is spread across multiple ponds (Voluntary Nature Conservancy, 2022) and experiences extremely low or no conflict. This is strikingly different from the conflict situation in Vadodara, where people have low tolerance towards muggers (Vyas and Stevenson, 2017), leading to high HMC. Of the 55 reported incidents in 2019–2023 from the entire state of Gujarat, 23 incidents (14 fatal and 9 non-fatal) took place in Vadodara, while Charotar had only two reported incidents (one fatal and one non-fatal). Both Charotar and Vadodara hold a significant population of mugger crocodiles (Vyas and Stevenson, 2017). However, due to the contrast in environmental (e.g. pollution due to sewage disposal, high degree of urbanization), ecological (e.g. lentic versus lotic habitats) and anthropogenic (e.g. the attitude of the locals, high incidence of HMC, greater dependency of the locals on water bodies) factors, muggers may exhibit varied physiological responses across the habitats.

Physiological responses and the overall well-being of free-ranging species have primarily been monitored via glucocorticoid metabolite (GCM) measurements. With high levels of anthropogenic disturbances and large-scale deterioration of habitats, most wildlife species experience varying levels of physiological stress (Millspaugh and Washburn, 2004), which is defined as the loss of homeostasis (Gaidica and Dantzer, 2020). Elevated levels of glucocorticoids for an extended period of time, chronic stress, have been shown to be detrimental to a wide range of taxa and cause significant negative impacts on the reproductive and immune systems (Blanchard *et al.*, 1995) of species. Due to the detrimental effects of chronic stress, conservation biologists across the globe use glucocorticoid measures to monitor the health and well-being of wildlife species (Martin *et al.,* 2018). Such monitoring provides a detailed understanding of whether the species is at risk under a given condition, and such findings have direct implications for conservation and management practices (Busch and Hayward, 2009). GCM can be measured from various types of substrates, for example hair, fur, urine, saliva and faeces (Cooke *et al.*, 2013). However, due to the ease of collection, faecal GCM (fGCM) measures have become largely popular. fGCM levels have been measured across diverse groups or taxa, including sungazer (S*maug giganteus*) (Scheun *et al.*, 2021), southern pied babblers (*Turdoides bicolor*) (Moagi *et al.*, 2021), southern yellow-billed hornbill (*Tockus leucomelas*) (Bouwer *et al.*, 2021), eastern rock sengis (*Elephantulus myurus*) (Medger *et al.*, 2021), Indian leopards (*Panthera pardus fusca*) (Panchal *et al.*, 2022), roan antelope (*Hippotragus equinus*) (Kamgang *et al.*, 2022), African elephant (*Loxodonta africana*) (Parker *et al.*, 2022) and rhesus macaques (*Macaca mulatta*) (Higham *et al.*, 2021), and have been validated and standardized as biomarkers for monitoring reproductive and immune functions of the species under diverse environmental conditions. fGCM monitoring allows for the assessment of both individual- and population-level characteristics without the need for capturing or handling animals, thus minimizing possible confounding effects on stress physiology. However, fGCM measurements can often be ambiguous if proper validation, treatment controls and systematic protocols are not followed, and interpretations can be complicated, particularly for cross-species comparisons (Millspaugh and Washburn, 2004; Busch and Hayward, 2009; Martin *et al.*, 2018; Romero and Beattie, 2022). Regardless of the challenges associated with fGCM measures, they have gained much popularity, primarily attributed to the ease of sample collection under natural or wild conditions. However, for the crocodilians, only two studies have been done so far to measure fGCM concentrations: one on Nile crocodiles (Ganswindt *et al.*, 2014) and the other on Cuban crocodiles (*Crocodylus rhombifer*) (Augustine *et al.*, 2020). To the best of our knowledge, fGCM measurements have not been conducted in muggers and, thus, need to be validated for the species.

Biological validation of a suitable assay system is crucial in order to evaluate the accuracy and reliability of the measured levels of the metabolites excreted in the faeces of a target species. Most species differ in steroid metabolism pathways, which, to some extent, is also influenced by the gut microflora. Thus, such differences cause these metabolites to vary across species (Touma and Palme, 2005). Therefore, biological validations become essential to ensure meaningful results from fGCM analysis (Heistermann *et al.,* 2006). Moreover, biological validation is preferred over physiological one as the latter requires invasive procedures, which are often logistically difficult to conduct on free-ranging organisms (Ganswindt *et al.*, 2014). Given this background, we developed the following objectives for our study: (i) Biological validation and standardization of an assay system to measure fGCM concentrations in mugger crocodiles, (ii) Application of the validated assay to free-ranging muggers, and compare and contrast fGCM levels in two diverse habitats, Vadodara (a mix of urban and rural habitats, mostly polluted due to industrial discharge and experience a high level of HMC) and Charotar (a rural agricultural and not an industrial area with low HMC, having high tolerance of locals towards the presence of muggers) regions within Gujarat in western part of India. We compared and contrasted fGCM data between Vadodara and Charotar regions during both breeding and non-breeding seasons of the muggers to assess if the levels differed between the two populations, and whether such variation was independent of the reproductive season of the species. Overall, this study aims to generate welfare indices, in terms of physiological readouts, for the free-ranging muggers inhabiting diverse ecological and environmental conditions within the tropical country of India.

## Materials and methods

### Biological validation using captive muggers

**Figure 2:**
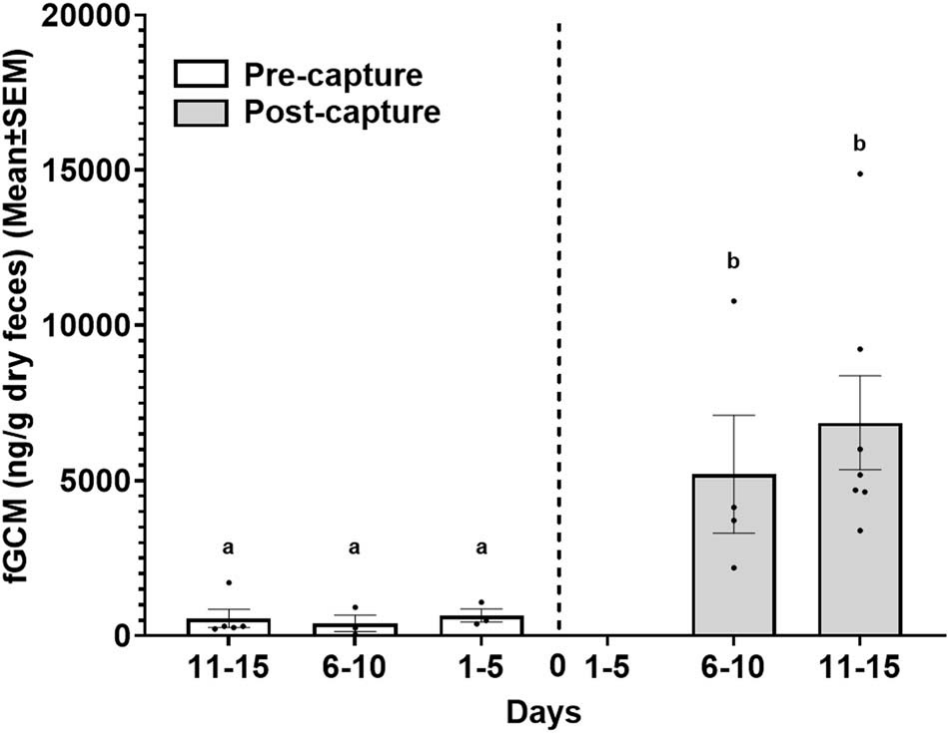
Levels of fGCM (mean ± SEM, ng/g dry faeces) in captive muggers during the biological validation study. Different alphabets represent significant differences (*P* ≤ 0.05).

Several studies have shown that restraint and capture can induce stress in most large vertebrates, activating the hypothalamic–pituitary–adrenal axis and leading to the secretion of glucocorticoids, indicative of stress (Mormède *et al.*, 2007). To biologically validate the suitability of a hormone assay system, we chose an enzyme immunoassay (EIA) targeting GCMs having 5β-3α-ol-11-one structure (11-oxoetiocholanolone EIA) (Palme and Möstl, 1997). The EIA was validated and successfully applied to the Nile crocodiles (Ganswindt *et al.*, 2014), belonging to the same genus as muggers. However, to validate the chosen assay for our target species, the mugger crocodiles, we conducted a study on a captive population of muggers in an enclosure housing 20 females and two males at the Madras Crocodile Bank Trust (MCBT) in Chennai, India. As a part of the annual medical checkup, we captured the muggers using a fish net to pull them out of the water and then secured them manually with ropes. Additionally, their snouts were taped, and gunny bags were placed over their head to prevent any potential damage to the eyes while being handled during the capture (Ebedes *et al.*, 2002). Each individual was restrained for ~15–20 minutes to assess health conditions by the veterinarian. All the muggers were housed in the same enclosure both before (pre-capture phase) and after (post-capture phase) the capture process. All the scats, found on the land, were collected from the enclosure 15 days before capture (*N* = 11 scats) and 15 days after capture (*N* = 11 scats). We considered day 0 to be the day of capture and plotted the fGCM concentrations for 1–5 days (*N* = 3 scats), 6–10 days (*N* = 3 scats) and 11–15 days (*N* = 5 scats) before capture. We followed a similar interval for the post-capture phase. However, there were no scats present on land for the 1–5 day period, whereas the 6–10 day and 11–15 day periods had a sample size of four and seven scats, respectively (Fig. 2). Since the enclosure had a large proportion of females when compared to males, the measured fGCM levels (both during pre and post-capture) might not reflect an equal representation of both the sexes.

### Study sites for free-ranging muggers

We collected scats from free-ranging mugger crocodiles across multiple sites within Charotar and Vadodara regions of Gujarat, India. The sampled sites (*N* = 13) were broadly classified into three different zones: A, B and C. Zone A (*N* = 5 sampling sites; Fig. 1) within Charotar, is a rural area, predominantly agricultural region with no industries and with local people having a high tolerance towards coexisting muggers, which mostly live in ponds or lakes and face low or no incidents of HMC (Voluntary Nature Conservancy, 2022). Zone B (*N* = 4 sampling sites; Fig. 1), an urban belt within Vadodara with high HMC (Vyas and Stevenson, 2017) and having several chemical industries disposing sewage in the rivers (e.g. the Vishwamitri river) (Bhangaonkar and Patel, 2017; Magadum *et al.*, 2017) that are predominantly mugger habitats (Vyas, 2013; Bhangaonkar and Patel, 2019). Zone C (*N* = 4 sampling sites; Fig. 1), a rural part within Vadodara having mainly agricultural lands and a low number of industries with high dependency of local people on adjoining rivers (e.g. the Vishwamitri river) that hold a significant mugger population (Vyas, 2013) (Fig. 1), and thus, having high HMC. Vishwamitri river cuts across both zones B (upstream sites) and C (downstream sites) (Fig. 1) and is a predominant mugger habitat.

### Scat collection procedure

For scat collection, each site was visited on multiple days over a period of 1 month during both breeding and non-breeding seasons. Re-visits were done typically after a gap of 2–5 days for a given site within a particular zone. Thus, pseudoreplication that is sampling the same individuals over time (across different calendar dates) for a particular site within a given zone could not be avoided during our study. Scats, found on land, were collected between 06:00 and10:00 h. for each site. Care was taken to avoid any close encounter with the large reptile. Fresh scats were collected in ziplock bags, and parameters like moisture content, smell and evidence of recent activity, e.g. fresh tracks, were used as a criterion to determine the freshness of the scat (Augustine *et al.*, 2020). Once a scat was sampled, the remaining portion was discarded to avoid confusion during the subsequent sampling round. Each scat sample was labelled, including the time of collection, location and date. Scats were stored in a cooler with ice packs at the field site and were moved to a freezer, maintaining −20°C within 5 hours of collection. All the samples were stored at −20°C until analysis. Muggers across the three zones, A, B and C, breed mainly from December to April (Whitaker and Whitaker, 1984). Thus, scat samples for the breeding season were collected during the month of December 2022; for the non-breeding data, we collected scats in June 2023. A total of 107 samples were collected during the breeding season (Zone A, *N* = 35; Zone B, *N* = 34; Zone C, *N* = 38), and 22 samples were collected during the non-breeding season (Zone A, *N* = 11; Zone B, *N* = 11). We could not collect any samples from Zone C during the non-breeding month of June because the sampled sites were flooded due to heavy rains. This was also the reason for having an overall lower sample size for non-breeding data on mugger crocodiles.

### Extraction and analysis of fGCM

**Figure 3:**
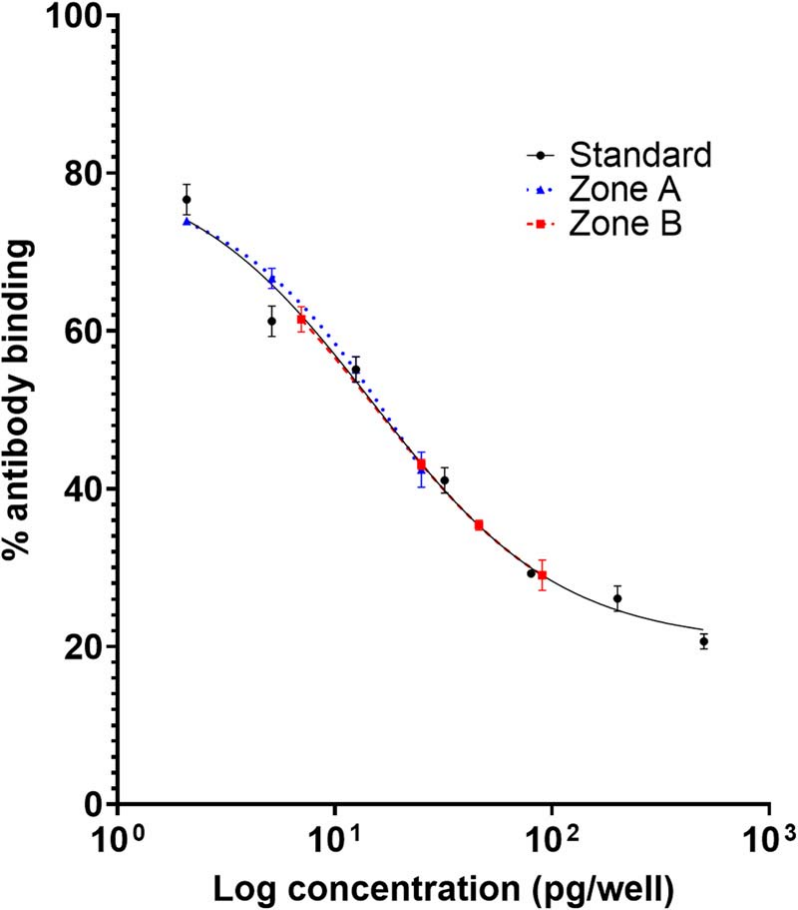
Parallelism plots showing serial dilution curves of faecal extracts for samples collected from free-ranging muggers, one from Zone A and one from zone B, and the standard curve.

All the collected scat samples were dried in a hot air oven at 80°C (Möstl *et al.*, 2005; Palme, 2005). Following drying, the scat samples were manually sieved and pulverized, and the dried faecal powder was stored at room temperature for further analysis (Panchal *et al.*, 2022). For extraction, 0.1 g of the dry scat powder was mixed with 3 ml of 80% methanol, vortexed for 3 minutes and then centrifuged at 1500 rpm for 10 minutes (Ganswindt *et al.*, 2014; Panchal *et al.*, 2022). After centrifugation, 2–2.5 ml of supernatant was collected and stored at −20°C for further analysis. fGCMs were quantified using a hormone assay targeting GCMs having 5β-3α-ol-11-one structure (11-oxoetiocholanolone EIA) and were purchased from Dr Rupert Palme (Department of Biomedical Sciences, University of Veterinary Medicine, Vienna, Austria). This specific assay was chosen to measure biologically relevant changes in the fGCM levels of mugger crocodiles. 11-oxo etiocholanolone was the target metabolite, and the assay used a standard curve ranging from 2.04 to 500 pg per well (Palme and Möstl, 1997; Möstl *et al.*, 2002). The details of cross reactivity is as follows: 5β-androstane-3α-ol-11,17-dione, 100%; 5β-pregnane-3α-ol-11,20-dione, 37%; 5β-androstane-3α,11β-diol-17-one, 3.3% and 5β-androstane-3,11,17-trione, 1.2%. All other tested cross-reactions were below 1% (11-ketoandrosterone, etiocholanolone, pregnanediol, tetrahydrocortisol, 5β-dihydro-cortisol, cortol 5β-pregnane-3α,11β,17α,20α,21-pentol, 5β-pregnane-3β-ol-11,20-dione and 5β-pregnane11β-diol-20-one) (Palme and Möstl, 1997). The assay was performed on anti-rabbit-IgG-coated (R2004, Sigma-Aldrich, USA) microtiter plates. The lowest concentration detected by the standard curve in terms of optical density (OD) values, defined as sensitivity of the assay, was determined at 2.04 pg per well. Concentrations below 2.04 pg per well resulted in similar OD values, thus, indicating that the standard curve is not sensitive to pick up changes in metabolite concentrations beyond this threshold. Each sample had four technical replicates, and we included two replicates in one single assay. The EC_50_ value, which represents the half-maximal effective concentration (mean ± SEM), was 55.2 ± 8.3 (*N* = 52 assays). Intra- and inter-assay coefficients of variation (CV) were 6.12% and 15.61%, respectively, calculated based on per well concentrations of a scat sample that served as an internal control for every assay performed. We also calculated CV for high (*N* = 10) and low (*N* = 10) fGCM containing samples using the measured OD values. The intra- and inter-assay CV was 8.95% and 12.31%, respectively, for low fGCM samples. The intra- and inter-assay CV was 5.93% and 14.84%, respectively, for high fGCM-containing samples. Faecal extracts of two of the samples of the free-ranging muggers, one from Zone A and another from Zone B, were serially diluted and assayed to check for parallelism with the standard curve (Fig. 3).

### Statistical analysis

The data were checked for normality using the Shapiro–Wilk test and variance was assessed via the *F*-test. If normality assumption was not met, the data were log-transformed. We used a linear mixed effects (LMEs) model to account for pseudoreplication as the same sites were visited over time for sampling scats. To assess effects of season (breeding and non-breeding) and zone (A, B, and C) on the fGCM (log-transformed) values of mugger crocodiles, we used the LME model (Bates *et al*. 2015) with the restricted maximum likelihood method. Each site within a given zone was repeatedly sampled for both the breeding and non-breeding seasons. Each sample received an ID and those collected on different dates but for the same site within a given zone had the same ID. Thus, ID was used as a random factor in the LME model. We did a parent LME model (Model1:logfGCM~season+zone, random = ~ 1|ID), which was compared with two other models (Model 2:logfGCM~season and Model 3:logfGCM~zone). Model comparisons (Model 1 versus Model 2 and Model 1 versus Model 3) using ANOVA were conducted to arrive at the best-fit model for the given data set. Apart from LME, *post hoc* analyses were done to conduct pairwise comparisons. For *post hoc* comparisons during the breeding season, one-way ANOVA with *post hoc* Tukey’s test was applied using fGCM as dependent and zones and ID as independent variables. For *post hoc* comparisons during the non-breeding season, Welch’s two-sample *t*-test was conducted to compare fGCM between zones A and B. To further check the effect of ID on the measured fGCM levels, we also conducted a separate one-way ANOVA including season (breeding and non-breeding), zone (A, B and C) and ID as independent variables and the measured fGCM levels (pooled across both breeding and non-breeding seasons) as dependent variable.

Parallel displacement between the standard curve and serial dilutions of the faecal extract was used to conduct analytical validation of the fGCM assay for the mugger crocodiles. The values within the linear range of the curve were subjected to linear regression analysis (PRISM software, version 9) using log concentration (pg/well) plotted against percent antibody binding of the standard and the sample dilution curves separately. The slopes of the regression lines were compared using the *F*-value of the regression analysis. All the statistical analyses were conducted using R version 4.2.3 using ‘nlme’, and ‘ggplot2’ packages, and the significance level was set at P < 0.05. All the reported data are presented as mean ± SEM and expressed as ng/g dry faeces.

## Results

### Parallelism curves

Slopes of the regression lines for the standard curve and the serially diluted samples (Fig. 3) were not significantly different (*F* = 2.09, *P* = 0.14). Thus, the assay confirms that it can be used for different free-ranging populations of the muggers.

### Biological validation

Biological validation results for the chosen assay showed an approximately 11-fold increase (*t* = 7.84, *P* = 8.126e−07, df = 15.72, Welch’s two-sample *t*-test) in fGCM levels (mean ± SEM) of captive muggers between pre-capture (540.9 ± 149.2 ng/g dry faeces, *N* = 11 samples collected 15 days before capture), and post-capture (6259.7 ± 1150.5 ng/g dry faeces, *N* = 11 samples collected till 15 days following capture) phases (Fig. 2). Further, fGCM levels during the pre-capture phase were 650.7 ± 215.9 ng/g dry faeces (*N* = 3) for 1–5 day period, 402.7 ± 264.6 ng/g dry faeces (*N* = 3) for 6–10 day period and 558.1 ± 287.6 ng/g dry faeces (*N* = 5) for 11–15 day period (Fig. 2). However, for post-capture phase, fGCM values were 5205.9 ± 1904.11 ng/g dry faeces (*N* = 4) for 6–10 day period and 6861.8 ± 1504.9 ng/g dry faeces, (*N* = 7) for 11–15 day period (Fig. 2). No scats were found during the 1–5 day period of the post-capture phase.

### fGCM levels in free-ranging muggers

**Figure 4:**
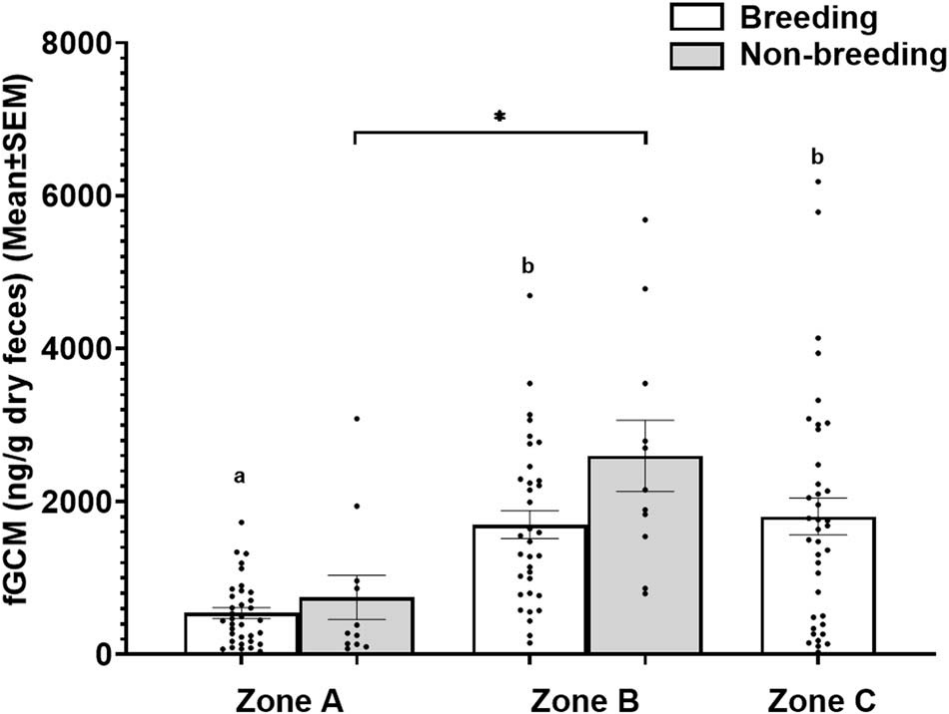
fGCM concentrations (mean ± SEM, ng/g dry faeces) of free-ranging mugger crocodiles during breeding and non-breeding seasons across the three zones, A, B and C. Different alphabets and asterisks indicate significant differences (*P* ≤ 0.05) across or between zones for breeding and non-breeding seasons, respectively.

The overall concentration of fGCM was 1412.7 ± 114.1 ng/g dry faeces (mean ± SEM, *n* = 129) for free-ranging muggers, pooled for both breeding and non-breeding seasons across all three zones. Comparison of the LME Model 1 (fgcm~season+zone) with that of the Model 2 (fgcm~season) showed significant (*P* < 0.05) differences (Table 1), however, with that of Model 3 (fgcm~zone) did not show any significant (*P* > 0.05) differences. Thus, season was excluded as a fixed effect and Model 3 was selected as the final LME model (Table 1), which showed a significant impact of zones on the measured fGCM levels. For pairwise comparisons, one-way ANOVA showed a significant (*P* < 0.05) effect of zones on the measured fGCM levels during the breeding season (Table 2), where Zone A (542.03 ± 71.3) was significantly (*P* < 0.05, *post hoc* Tukey’s test) different from Zone B (1699.9 ± 180.8) and Zone C (1806.4 ± 243.2) (Fig. 4). However, no significant (*P* > 0.05, *post hoc* Tukey test) differences were observed in fGCM levels between zones B and C during the breeding season of the muggers (Fig. 4). For the non-breeding season data, significant (*P* < 0.00072, *t* = −4.2441 Welch’s two sample *t*-test) differences were observed between Zone A (747.8 ± 288.8) and Zone B (2600.5 ± 465.9) (Fig. 4).

**Table 1:** Results of LME model and comparison of models using ANOVA

**LME Model 1**							
Random effects:							
Formula: ~1|ID							
Intercept: Zone A							
Fixed effects: logfGCM~season+zone							
	**Value**	**Std. error**	**df**	***t*** **-value**	***P*** ** value**		
**Intercept**	2.67	0.10	79	24.41	0.0000		
**seasonR**	−0.094	0.10	79	−0.93	0.35		
**Zone B**	0.57	0.11	46	4.85	0.0000		
**Zone C**	0.40	0.11	46	3.40	0.001		
**LME Model 2**							
Random effects:							
Formula: ~1|ID							
Intercept: Zone A							
Fixed effects: logfGCM~season							
	**Value**	**Std. error**	**df**	***t*** ** value**	***P*** ** value**		
**Intercept**	2.94	0.10	79	27.92	0.0000		
**seasonR**	−0.07	0.10	79	−0.71	0.47		
**LME Model 3**							
Random effects:							
Formula: ~1|ID							
Intercept: Zone A							
Fixed effects: logfGCM~zone							
	**Value**	**Std. error**	**df**	***t*** ** value**	***P*** ** value**		
**Intercept**	2.60	0.077	80	33.83	0.0000		
**Zone B**	0.57	0.11	46	4.83	0.0000		
**Zone C**	0.37	0.11	46	3.25	0.0021		
**Comparison of Model 1 versus Model 2**							
**Model**	**df**	**AIC**	**BIC**	**LogLik**	**Test**	**L.Ratio**	***P* value**
1	6	171.47	188.44	−79.73			
2	4	181.37	192.74	−86.68	1 vs. 2	13.89	0.001
Model 1: lme (logfGCM~season+zone,(1|ID))							
Model 2: lme (logfGCM~season,(1|ID))							
**Comparison of Model 1 versus Model 3**							
**Model**	**df**	**AIC**	**BIC**	**LogLik**	**Test**	**L.Ratio**	***P* value**
1	6	171.47	188.44	−79.73			
3	5	167.59	181.77	−78.79	1 vs. 2	1.87	0.17
Model 1: lme(logfGCM~season+zone,(1|ID))							
Model 3: lme(logfGCM~zone,(1|ID))							

**Table 2:** One-way ANOVA with *post hoc* Tukey’s test showing the impact of zones and ID on fGCM levels during the breeding season

	**Estimate**	**Std. error**	***t*-value**	**Pr(>|z|)**
**Intercept**	2.63	0.088	29.82	< 2e-16
**ZoneB**	1.03	0.37	2.75	0.006
**ZoneC**	0.75	0.23	3.14	0.002
**ID**	−0.005	0.004	−1.33	0.18
**Post hoc Tukey’s test**				
**Zone**	**Estimate**	**Std. error**	**Z value**	**Pr(>|z|)**
**B–A**	1.03	0.37	2.75	0.013
**C–A**	0.75	0.23	3.14	0.004
**C–B**	−0.27	0.17	−1.58	0.19

The one-way ANOVA conducted using ID as a fixed effect showed significant (*P* < 0.05) impact of only zones on the measured fGCM levels, and no significant (*P* > 0.05) results were obtained for seasons and ID (Table 3).

**Table 3:** One-way ANOVA showing the effect of season, zone, and ID on fGCM levels pooled across both breeding and non-breeding seasons

	**Estimate**	**Std. error**	***t*-value**	**Pr(>|z|)**
**Intercept**	2.69	0.12	21.45	< 2e-16
**seasonR**	−0.11	0.11	−1.08	0.28
**Zone B**	0.80	0.31	2.51	0.01
**Zone C**	0.60	0.20	2.89	0.004
**ID**	−0.002	0.003	−0.63	0.52

## Discussion

The study biologically validated an assay targeting GCMs having 5β**-**3α-ol-11-one structure (11-oxoetiocholanolone EIA) (Palme and Möstl, 1997) for mugger crocodiles by showing a significant difference (11-fold) in fGCM levels between the pre- and post-capture phases of the captive muggers. The study also demonstrated that fGCM levels were significantly different between Charotar (Zone A) and Vadodara (Zones B and C), and the pattern was similar during both the breeding and non-breeding seasons of the mugger crocodiles. During the breeding season, Zone A in Charotar showed a significantly lower level of fGCM compared to fGCM concentrations in zones B and C, both occurring in Vadodara. A similar contrast was maintained during the non-breeding season, where Zone A in Charotar again had significantly lower fGCM concentrations than Zone B in Vadodara. Thus, muggers in Vadodara had high fGCM levels during both breeding (sampled during December 2022) and non-breeding (sampled during June 2023) seasons, possibly indicating a state of chronic stress.

Capture stress is a popular method to biologically validate fGCM measures in a species and has been applied to a range of different organisms, for example, lace monitors (*Varanus varius*) (Scheelings and Jessop, 2011), stump-tailed macaques (*Macaca arctoides*) (Pineda-Galindo *et al.*, 2017), Eurasian red squirrels (*Sciurus vulgaris*) (Dantzer *et al.*, 2016), yellow-bellied marmots (*Marmota flaviventris*) (Smith *et al.*, 2012), polar bears (*Ursus maritimus*) (Hein *et al.*, 2020) and African penguins (*Spheniscus demersus*) (Driscoll *et al.*, 2023). With regard to experimental logistics, biological validation, either via capture and restraint (Smith *et al.*, 2012), physical injury (Cope *et al.*, 2022) or disease (Laver *et al.*, 2012) and distress (Hein *et al.*, 2020), has become a chosen method to standardize an EIA targeting GCMs. This is mainly because the alternative method, which is performing an adrenocorticotropic hormone stimulation test (ACTH challenge test) (Laver *et al.*, 2012; Terwissen *et al.*, 2013; Ganswindt *et al.*, 2014) to validate EIAs, requires injecting synthetic ACTH products into an organism and is thus logistically challenging to perform when compared to biological validation tests. As a part of this study and the previous study by Ganswindt *et al.* (2014), the same 11-oxoetiocholanolone EIA has now been validated for Nile and mugger crocodile species, both belonging to the same genus. In our study, fGCM levels (pre-capture, 540.9 ± 149.2 ng/g dry faeces) measured in the captive mugger crocodiles were similar to the levels (pre-challenge test, 690 ± 100 ng/g dry faecal powder) measured in farm-raised Nile crocodiles (Ganswindt *et al.*, 2014), and both studies used the same EIA. Although our study may have included repeat sampling from the same individuals over time (due to a lack of focal individual identification), the application of a single validated EIA will facilitate generating robust cross-species comparisons, including global populations of the two crocodile species living under varied conditions (Martin, 2008; Mobaraki *et al.*, 2021; Desai *et al.*, 2022). Such a comparative approach will serve as a great asset, contributing to the field of wildlife endocrinology (Ghosal *et al.*, 2023).

Our study revealed that the captive (pre-capture) mugger crocodiles at MCBT had similar levels of fGCM as the free-ranging muggers in Charotar (Zone A). Contrastingly, fGCM levels of muggers in Vadodara (zones B and C) were four times higher compared to both the captive (pre-capture) and Charotar populations. A study on yellow-bellied marmots (Smith *et al.*, 2012) demonstrated that fGCM levels in the free-ranging population were 68% higher than in the captive population. This suggests that free-ranging marmots experience a more challenging environment in the presence of predators and face high levels of inter-individual competition for limited resources, compared to well-fed captive marmots (Smith *et al.*, 2012). This contrasts with our findings on captive mugger crocodiles and the free-ranging muggers in Charotar, both of which had similar fGCM levels. The studbook record of MCBT showed that the captive muggers included in our study are healthy, as determined during the annual medical checkups by the zoo veterinarian, and the crocodiles have been breeding successfully (in terms of viable eggs) for the past two decades (Whitaker and Whitaker, 1984; Desai *et al.*, 2022). Thus, it can be speculated that muggers in Charotar (a free-ranging natural condition) are facing less challenging situations compared to muggers in Vadodara and are possibly experiencing physiological conditions similar to those of the captive muggers at MCBT. In contrast, Vadodara muggers have significantly high fGCM levels, which could either represent a compromised, maladaptive condition (Angelier, 2022; Forgione and Brady, 2022) or an adaptive physiological response towards a highly challenging environment (Boonstra, 2013; Geffroy and Douhard, 2019).

Stress hormones or glucocorticoids (GCs) are released in the body to mobilize energy to combat challenging conditions and are thus adaptive in nature. However, long-term maintenance of elevated GC levels is detrimental to the fitness of the organism and has severe consequences, including reproductive failure and poor immune function (Schoech *et al.*, 1991; Blanchard *et al.*, 1995). Several studies, for example, by Romero and Wikelski (2001), Clinchy *et al.* (2004), Kitaysky *et al.* (2010) and Boonstra (2013), have argued against that and claimed that chronic stress may not always be detrimental (in terms of fitness) for an organism that lives under natural, free-ranging conditions. Natural conditions are far more challenging in terms of both ecological and environmental conditions compared to laboratory conditions or captivity. However, most of the research demonstrating adverse effects of long-term stress (chronic stress) has been associated with laboratory findings (Rice *et al.*, 2008; Elizalde *et al.*, 2010), and several studies argue that such findings cannot be directly translated to free-ranging conditions, as the animals in the wild are exposed to a wide variety of stressors and their physiological systems would have developed a range of adaptive solutions to cope with such ecological and environmental problems (Wingfield, 2013; Hawkins and Storey, 2020). Thus, in this study, whether the high fGCM levels in muggers of Vadodara (zones B and C) indicate a potential maladaptive condition of chronic stress or a physiological adaptation of the population under a severely challenged condition or may represent natural variations across populations warrants further investigations. Future studies should include additional well-being measures, for example behavioural measures to determine basking patterns that maintain homeostasis (Rusch and Angilletta Jr, 2017) or measures of nutritional health in terms of thyroid hormone levels (Schaebs *et al.*, 2016) to investigate whether high fGCM concentrations in Vadodara muggers indicate a ‘stressful’ condition or an ‘ecophysiological adaptation’ of the population.

The breeding phase in animals has often been associated with high levels of GC when compared to non-breeding phases, and this is mainly because courtship, mate choice and mating are energetically expensive processes, and investing in such processes is directly linked to the fitness of the organisms (Romero, 2002; Balestri *et al.*, 2014; Rudolph *et al.*, 2020). However, this does not seem to be the case for the mugger crocodiles in our study. The contrast in fGCM levels of muggers between Charotar and Vadodara was similar during both breeding and non-breeding seasons. Thus, it can be speculated that the fGCM responses of the sampled muggers are mainly influenced by the local environment rather than by the individuals’ biological (breeding or non-breeding) state. Similar results have been obtained for several species (Weingrill *et al.*, 2004; Kitaysky *et al.*, 2007), for example the seabird, Common Murre (*Uria aalge*), which showed that corticosterone secretion was strongly correlated with environmental conditions, for example food abundance, and did not show much association with the reproductive stage of the birds (Kitaysky *et al.,* 2007). We speculate that high fGCM levels in muggers of Vadodara (Zone B) could be due to a combination of different factors, including habitat degradation due to pollution (Magadum *et al.*, 2017; Bhangaonkar and Patel, 2019), a low tolerant attitude of locals towards muggers and a high dependency on water bodies leading to high HMC (Vyas and Stevenson, 2017) and encroachment of habitats due to urbanization (Meena *et al.*, 2023). Although we did not measure the level of pollutants in the water bodies in this study, the presence of a large number of industries in Vadodara and the contaminants measured in the Vishwamitri River (which is also our study site, zones B and C) in recent studies by Magadum *et al.* (2017) and Bhangaonkar and Patel (2019) support the speculation that fGCM levels in Vadodara muggers may potentially be also influenced by the level of pollution in their respective habitats. Moreover, the pollutants (Pb, Fe, Hg, Cd, Ni, Cu, Mn, Zn) detected in the Vishwamitri River (Magadum *et al.*, 2017; Bhangaonkar and Patel, 2019) have been shown in several studies to adversely impact the health of a species. For example, studies on the southern toad (*Bufo terrestris*), common carp (*Labeo rohita*) and white storks (*Ciconia ciconia*) showed that the level of pollutants (Cd, Cu, Pb, Zn) influenced fGCM levels in the populations and, in turn, reduced the survival and reproductive success of the species (Baos *et al.*, 2006; Busch and Hayward, 2009; Sumera *et al.*, 2018). Future research on muggers should aim to investigate the reproductive biology (e.g. measuring reproductive hormones, clutch size and eggshell thickness) of the individuals living in such polluted sites, for example the Vishwamitri River of Vadodara, to determine the effect of contaminants on the overall fitness of the given population.

In a free-ranging population, it is difficult to investigate a single cause behind the measured physiological responses, high or low levels of fGCM. However, inclusion of multiple variables, for example habitat quality, measure of anthropogenic disturbances, ethnographic measure of human attitudes towards co-existing wildlife species, reproductive health (in terms of behaviour, endocrine correlates, clutch size and eggshell thickness), diet and social rank of individuals, will facilitate robust correlations with the fGCM levels and provide significant insights in characterizing plausible explanations for such responses (Martínez-Mota *et al.*, 2016; Harrison *et al.*, 2021). Besides these factors, as mentioned in the studies by Busch and Hayward (2009) and Romero and Beattie (2022), several other factors, for example temperature, pathogen load and density of individuals, need to be considered before developing informed strategies based on fGCM measurements for conservation and management of the target species (Dantzer *et al.,* 2014). In conclusion, our study showed that physiological responses, in terms of fGCM levels, varied across populations for a large freshwater reptilian species. To the best of our knowledge, this is the first study that has non-invasively monitored fGCM levels in a free-ranging crocodilian species. The study highlights the need for long-term future research to investigate whether the heightened levels of fGCM in Vadodara populations represent chronic stress or an adaptive trait in the vulnerable mugger species.

## Data Availability

All the data are available in the manuscript’s figures.

## References

[ref1] Angelier F (2022) Consequences of developmental exposure to pollution: importance of stress-coping mechanisms. In D Costantini, V Marasco, eds, Development Strategies and Biodiversity: Darwinian Fitness and Evolution in the Anthropocene. Springer International Publishing, Cham, pp. 283–316.

[ref2] Aresco MJ (2005) Mitigation measures to reduce highway mortality of turtles and other herpetofauna at a North Florida lake. J Wildl Manag 69: 549–560. 10.2193/0022-541X(2005)069[0549:MMTRHM]2.0.CO;2.

[ref3] Augustine L , Miller K, Peters A, Franklin AD, Steinbeiser CM, Brown JL, Prado NA (2020) Impacts of the season and reproductive status on fecal reproductive and adrenocortical steroid metabolites in zoo Cuban crocodiles (*Crocodylus rhombifer*). Zoo Biol 39: 411–421. 10.1002/zoo.21559. 32770706

[ref4] Balestri M , Barresi M, Campera M, Serra V, Ramanamanjato JB, Heistermann M, Donati G (2014) Habitat degradation and seasonality affect physiological stress levels of *Eulemur collaris* in littoral forest fragments. PloS One 9: e107698. 10.1371/journal.pone.0107698. 25229944 PMC4168001

[ref5] Baos R , Blas J, Bortolotti GR, Marchant TA, Hiraldo F (2006) Adrenocortical response to stress and thyroid hormone status in free-living nestling white storks (*Ciconia ciconia*) exposed to heavy metal and arsenic contamination. Environ Health Perspect 114: 1497–1501. 10.1289/ehp.9099. 17035132 PMC1626439

[ref63] Bates D , Mächler M, Bolker B, Walker S (2015) Fitting Linear Mixed-Effects Models Using lme4. Journal of Statistical Software 67: 1–48. 10.18637/jss.v067.i01.

[ref6] Bhangaonkar PD , Patel JS (2017) Water quality zoning of Vishwamitri river to access environmental flow requirements through aggregation of water quality index. Int J Hum Cap Urban Manag 4: 281–292. 10.22034/ijhcum.2017.02.04.004.

[ref7] Bhangaonkar PD , Patel JS (2019) Assessment of heavy metals in surface water of Vishwamitri River. Int J Hort Sci Technol 9: 675–689. 10.1504/IJHST.2019.103449.

[ref8] Bhowmik S , Bhatt B (2023) Spatiotemporal analysis of land surface temperature owing to NDVI: a case study of Vadodara District, Gujarat. J Geomat 17: 48–57. 10.58825/jog.2023.17.1.83.

[ref9] Blanchard DC , Spencer RL, Weiss SM, Blanchard RJ, McEwen B, Sakai RR (1995) Visible burrow system as a model of chronic social stress: behavioral and neuroendocrine correlates. Psychoneuroendocrinology 20: 117–134. 10.1016/0306-4530(94)E0045-B. 7899533

[ref10] Boonstra R (2013) The ecology of stress: a marriage of disciplines. Funct Ecol 27: 7–10. 10.1111/1365-2435.12048.

[ref11] Bouwer M , Ngcamphalala CA, Ganswindt A, McKechnie AE (2021) Validation of a non-invasive technique for quantifying a stress-associated biomarker in a southern African hornbill. J Ornithol 162: 615–619. 10.1007/s10336-021-01861-5.

[ref12] Brackhane S , Webb G, Xavier FM, Gusmao M, Pechacek P (2018) When conservation becomes dangerous: human-crocodile conflict in Timor-Leste. J Wildl Manag 82: 1332–1344. 10.1002/jwmg.21497.

[ref13] Brien ML , Gienger CM, Browne CA, Read MA, Joyce MJ, Sullivan S (2017) Patterns of human–crocodile conflict in Queensland: a review of historical estuarine crocodile (*Crocodylus porosus*) management. Wildl Res 44: 281–290. 10.1071/WR17011.

[ref14] Buenfil-Rojas AM , Alvarez-Legorreta T, González-Jáuregui M, Rendón-von Osten J, Cedeño-Vazquez JR (2022) Effectiveness of Morelet's crocodile as a bioindicator of metal pollution and metallothionein response to spatial variations of metal exposure. Environ Adv 8: 100251. 10.1016/j.envadv.2022.100251.

[ref15] Busch DS , Hayward LS (2009) Stress in a conservation context: a discussion of glucocorticoid actions and how levels change with conservation-relevant variables. Biol Conserv 142: 2844–2853. 10.1016/j.biocon.2009.08.013.

[ref16] Cavalier R , Pratt EN, Serenari C, Rubino EC (2022) Human dimensions of crocodilians: a review of the drivers of coexistence. Hum Dimens Wildl 27: 380–396. 10.1080/10871209.2021.1953195.

[ref17] Choudhury BC , De Silva A (2013) Crocodylus palustris. The IUCN Red List of Threatened Species, IUCN, Switzerland.

[ref18] Clinchy M , Zanette L, Boonstra R, Wingfield JC, Smith JN (2004) Balancing food and predator pressure induces chronic stress in songbirds. Proc Biol Sci 271: 2473–2479. 10.1098/rspb.2004.2913. 15590598 PMC1691882

[ref19] Cooke SJ , Sack L, Franklin CE, Farrell AP, Beardall J, Wikelski M, Chown SL (2013) What is conservation physiology? Perspectives on an increasingly integrated and essential science. Conserv Physiol 1: cot001. 10.1093/conphys/cot001. 27293585 PMC4732437

[ref20] Cope HR , Keeley T, Keong J, Smith D, Silva FR, McArthur C, Webster KN, Mella VSA, Herbert CA (2022) Validation of an enzyme immunoassay to measure faecal glucocorticoid metabolites in common brushtail possums (*Trichosurus vulpecula*) to evaluate responses to rehabilitation. J Anim 12: 1627. 10.3390/ani12131627. PMC9265043 35804526

[ref21] Corvera MD , Manalo RI, Aquino MTR (2017) People and crocodiles sharing one environment: an analysis of local human-crocodile conflict management strategies in the Philippines. J Anim Sci 1: 1–6. 10.16966/jasr.105.

[ref22] Dantzer B , Fletcher QE, Boonstra R, Sheriff MJ (2014) Measures of physiological stress: a transparent or opaque window into the status, management and conservation of species? Conserv Physiol 2: cou023. 10.1093/conphys/cou023. 27293644 PMC4732472

[ref23] Dantzer B , Santicchia F, van Kesteren F, Palme R, Martinoli A, Wauters LA (2016) Measurement of fecal glucocorticoid metabolite levels in Eurasian red squirrels (*Sciurus vulgaris*): effects of captivity, sex, reproductive condition, and season. J Mammal 97: 1385–1398. 10.1093/jmammal/gyw095.

[ref24] Das CS , Jana R (2018) Human–crocodile conflict in the Indian Sundarban: an analysis of spatio-temporal incidences in relation to people's livelihood. Oryx 52: 661–668. 10.1017/S0030605316001502.

[ref25] Desai B , Patel A, Patel V, Shah S, Raval MS, Ghosal R (2022) Identification of free-ranging mugger crocodiles by applying deep learning methods on UAV imagery. Ecol Inform 72: 101874. 10.1016/j.ecoinf.2022.101874.

[ref26] Desai R (2017) Entitlements of seasonal migrant construction workers to housing, basic services and social infrastructure in Gujarat’s cities: background policy paper. Centre for Urban Equity Working Paper 35: 101874.

[ref27] Driscoll MV , Tuttle AD, Romano TA (2023) Fecal glucocorticoid analysis as a health monitoring tool for endangered African penguins (*Spheniscus demersus*). Gen Comp Endocrinol 330: 114147. 10.1016/j.ygcen.2022.114147. 36272448

[ref28] Ebedes H , Van Rooyen J, Du Toit JG (2002) Capturing wild animals. In JDP Bothma, ed, Game Ranch Management, 4th ed. Van Schaik Uitgewers, Pretoria, pp. 382–430.

[ref29] Elizalde N , García-García AL, Totterdell S, Gendive N, Venzala E, Ramirez MJ, Del Rio J, Tordera RM (2010) Sustained stress-induced changes in mice as a model for chronic depression. Psychopharmacology (Berl) 210: 393–406. 10.1007/s00213-010-1835-6. 20401750

[ref30] Forgione ME , Brady SP (2022) Road salt is more toxic to wood frog embryos from polluted ponds. Environ Pollut 296: 118757. 10.1016/j.envpol.2021.118757. 34973378

[ref31] Fukuda Y , Manolis C, Appel K (2014) Featured article: management of human-crocodile conflict in the Northern Territory, Australia: review of crocodile attacks and removal of problem crocodiles. J Wildl Manag 78: 1239–1249. 10.1002/jwmg.767.

[ref32] Gaidica M , Dantzer B (2020) Quantifying the autonomic response to stressors—one way to expand the definition of “stress” in animals. Integr Comp Biol 60: 113–125. 10.1093/icb/icaa009. 32186720

[ref33] Ganswindt SB , Myburgh JG, Cameron EZ, Ganswindt A (2014) Non-invasive assessment of adrenocortical function in captive Nile crocodiles (Crocodylus niloticus). Comp Biochem Physiol A Mol Integr Physiol 177: 11–17. 10.1016/j.cbpa.2014.07.013. 25066028

[ref34] García-Grajales J , Buenrostro-Silva A (2019) Assessment of human–crocodile conflict in Mexico: patterns, trends and hotspots areas. Mar Freshw Res 70: 708–720. 10.1071/MF18150.

[ref35] Geffroy B , Douhard M (2019) The adaptive sex in stressful environments. Trends Ecol Evol 34: 628–640. 10.1016/j.tree.2019.02.012. 30952545

[ref36] Ghosal R , Edwards KL, Chiarelli TL, Fanson KV, Ganswindt A, Keeley T, Koester DC, Roberts B, Majelantle TL, Wauters J et al. (2023) Biomarkers of reproductive health in wildlife and techniques for their assessment. Theriogenology Wild 3: 100052. 10.1016/j.therwi.2023.100052.

[ref37] Grigg G , Gans C (1993) Morphology & Physiology of the Crocodylia. editors. Fauna of Australia, 2A: Amphibia and Reptilia. Australian Government Publishing Service, Canberra, pp. 326–336.

[ref38] Guillette LJ Jr , Pickford DB, Crain DA, Rooney AA, Percival HF (1996) Reduction in penis size and plasma testosterone concentrations in juvenile alligators living in a contaminated environment. Gen Comp Endocrinol 101: 32–42. 10.1006/gcen.1996.0005. 8713642

[ref39] Harrison ND , Maag N, Haverkamp PJ, Ganswindt A, Manser MB, Clutton-Brock TH, Ozgul A, Cozzi G (2021) Behavioural change during dispersal and its relationship to survival and reproduction in a cooperative breeder. J Anim Ecol 90: 2637–2650. 10.1111/1365-2656.13569. 34258771 PMC8597146

[ref40] Hawkins LJ , Storey KB (2020) Advances and applications of environmental stress adaptation research. Comp Biochem Physiol A Mol Integr Physiol 240: 110623. 10.1016/j.cbpa.2019.110623. 31778815

[ref41] Hein A , Palme R, Baumgartner K, von Fersen L, Woelfing B, Greenwood AD, Bechsoft T, Siebert U (2020) Faecal glucocorticoid metabolites as a measure of adrenocortical activity in polar bears (*Ursus maritimus*). Conser physiol 8: coaa012. 10.1093/conphys/coaa012. PMC7125046 32274062

[ref42] Heistermann M , Palme R, Ganswindt A (2006) Comparison of different enzyme immunoassays for assessment of adrenocortical activity in primates based on fecal analysis. Am J Primatol 68: 257–273. 10.1002/ajp.20222. 16477600

[ref43] Henkanaththegedara SM , Sideleau B, Anwar Y, Haidir IA, Amarasinghe AT (2023) Integrating social and ecological information to identify high-risk areas of human-crocodile conflict in the Indonesian archipelago. Biol Conserv 280: 109965. 10.1016/j.biocon.2023.109965.

[ref44] Higham JP , Kimock CM, Mandalaywala TM, Heistermann M, Cascio J, Petersdorf M, Winters S, Allen WL, Dubuc C (2021) Female ornaments: is red skin color attractive to males and related to condition in rhesus macaques? Behav Ecol 32: 236–247. 10.1093/beheco/araa121. 33814977 PMC7995641

[ref45] Humphries MS , Myburgh JG, Campbell R, Buah-Kwofie A, Combrink X (2021) Organochlorine pesticide bioaccumulation in wild Nile crocodile (*Crocodylus niloticus*) fat tissues: environmental influences on changing residue levels and contaminant profiles. Sci Total Environ 753: 142068. 10.1016/j.scitotenv.2020.142068. 32911174

[ref46] Kamgang VW , Bennett NC, van der Goot AC, Majelantle TL, Ganswindt A (2022) Patterns of faecal glucocorticoid metabolite levels in captive roan antelope (*Hippotragus equinus*) in relation to reproductive status and season. Gen Comp Endocrinol 325: 114052. 10.1016/j.ygcen.2022.114052. 35568230

[ref47] Khan W , Hore U, Mukherjee S, Mallapur G (2020) Human-crocodile conflict and attitude of local communities toward crocodile conservation in Bhitarkanika wildlife sanctuary, Odisha, India. Mar Policy 121: 104135. 10.1016/j.marpol.2020.104135.

[ref48] Kitaysky AS , Piatt JF, Hatch SA, Kitaiskaia EV, Benowitz-Fredericks ZM, Shultz MT, Wingfield JC (2010) Food availability and population processes: severity of nutritional stress during reproduction predicts survival of long-lived seabirds. Funct Ecol 24: 625–637. 10.1111/j.1365-2435.2009.01679.x.

[ref49] Kitaysky AS , Piatt JF, Wingfield JC (2007) Stress hormones link food availability and population processes in seabirds. Mar Ecol Prog Ser 352: 245–258. 10.3354/meps07074.

[ref50] Kumar S , Goel NK (2014) Analysis of non-stationarity in hydro-meteorological data of Dhadhar River basin. Int J Eng Res Technol Conf Proc 3: 2–5.

[ref51] Laver PN , Ganswindt A, Ganswindt SB, Alexander KA (2012) Non-invasive monitoring of glucocorticoid metabolites in banded mongooses (*Mungos mungo*) in response to physiological and biological challenges. Gen Comp Endocrinol 179: 178–183. 10.1016/j.ygcen.2012.08.011. 22926328

[ref52] Magadum A , Patel T, Gavali D (2017) Assessment of physicochemical parameters and water quality index of Vishwamitri River, Gujarat, India. Int J Environ Agric Biotechnol 2: 1505–1510. 10.22161/ijeab/2.4.8.

[ref53] Makwana RH (2017) Emerging youth leadership in panchayat of Gujarat. Delib Res 33: 61.

[ref54] Maphanga T , Madonsela BS, Chidi BS, Shale K, Munjonji L, Lekata S (2022) The effect of rainfall on *Escherichia coli* and chemical oxygen demand in the effluent discharge from the Crocodile River wastewater treatment; South Africa. Water 14: 2802. 10.3390/w14182802.

[ref55] Martin LB , Vitousek M, Donald JW, Flock T, Fuxjager MJ, Goymann W, Hau M, Husak J, Johnson MA, Kircher B et al. (2018) IUCN conservation status does not predict glucocorticoid concentrations in reptiles and birds. Integr Comp Biol 58: 800–813. 10.1093/icb/icy102. 30052988

[ref56] Martin S (2008) Global diversity of crocodiles (Crocodilia, Reptilia) in freshwater. Springer Netherlands, Dordrecht, Netherlands, pp. 587–591.

[ref57] Martínez-Mota R , Righini N, Palme R (2016) Fluctuations in daily energy intake do not cause physiological stress in a Neotropical primate living in a seasonal forest. Oecologia 182: 973–984. 10.1007/s00442-016-3739-6. 27681556

[ref58] Medger K , Prins A, Lutermann H, Ganswindt A, Ganswindt SB, Bennett NC (2021) Repeatability of daily profiles of baseline glucocorticoid metabolites measured in the urine and faeces of eastern rock sengis (*Elephantulus myurus*). Gen Comp Endocrinol 312: 113857. 10.1016/j.ygcen.2021.113857. 34284023

[ref59] Meena H , Manekar VL, Patel JN (2024) Effect of land use land cover changes on urban floods check for updates. In Flood Forecasting and Hydraulic Structures: Proceedings of 26th International Conference on Hydraulics, Water Resources and Coastal Engineering (HYDRO 2021). Springer Nature, Singapore, pp. 245–252.

[ref60] Millspaugh JJ , Washburn BE (2004) Use of fecal glucocorticoid metabolite measures in conservation biology research: considerations for application and interpretation. Gen Comp Endocr 138: 189–199. 10.1016/j.ygcen.2004.07.002. 15364201

[ref61] Moagi LL , Bourne AR, Cunningham SJ, Jansen R, Ngcamphalala CA, Ganswindt A, Ridley AR, McKechnie AE (2021) Hot days are associated with short-term adrenocortical responses in a southern African arid-zone passerine bird. J Exp Biol 224: jeb242535. 10.1242/jeb.242535. 34032270

[ref62] Mobaraki A , McCaskill L, Schepp U, Abtin E, Masroor R, Pandhi D, Desai B, Mukherjee S, Rasheed T, Razzaque SA et al. (2021) Conservation status of the mugger (*Crocodylus palustris*): establishing a working group for a poster species of climate change. IUCN- Crocodile Specialist Group (CSG) Newsletter 40: 12–20 http://www.iucncsg.org/.

[ref64] Mormède P , Andanson S, Aupérin B, Beerda B, Guémené D, Malmkvist J, Manteca X, Manteuffel G, Prunet P, van Reenen CG et al. (2007) Exploration of the hypothalamic–pituitary–adrenal function as a tool to evaluate animal welfare. Physiol Behav 92: 317–339. 10.1016/j.physbeh.2006.12.003. 17234221

[ref65] Möstl E , Maggs JL, Schrötter G, Besenfelder U, Palme R (2002) Measurement of cortisol metabolites in faeces of ruminants. Vet Res Commun 26: 127–139. 10.1023/A:1014095618125. 11922482

[ref66] Möstl E , Rettenbacher S, Palme R (2005) Measurement of corticosterone metabolites in birds' droppings: an analytical approach. Ann N Y Acad Sci 1046: 17–34. 10.1196/annals.1343.004. 16055841

[ref67] Oommen MA (2021) Beasts in the garden: human-wildlife coexistence in India's past and present. Front Conserv Sci 2: 703432. 10.3389/fcosc.2021.703432.

[ref68] Pagdand (2019) Report on Mugger Crocodile Countat Vishwamitri, Vadodara, Gujarat, India: With an aim of Wildlife Conservation, Pagdand, Vadodara, Gujarat, India, p. 42.

[ref69] Palme R (2005) Measuring fecal steroids: guidelines for practical application. Ann N Y Acad Sci 1046: 75–80. 10.1196/annals.1343.007. 16055844

[ref70] Palme R , Möstl E (1997) Measurement of cortisol metabolites in faeces of sheep as a parameter of cortisol concentration in blood. Int J Mamm Biol 62: 192–197.

[ref71] Panchal N , Desai C, Ghosal R (2022) Fecal glucocorticoid metabolite levels in captive Indian leopards (*Panthera pardus fusca*) housed under three different enrichment regimes. PloS One 17: e0261796. 10.1371/journal.pone.0261796. 36083970 PMC9462577

[ref72] Panchayat, Rural housing, and Rural development department, Government of Gujarat panchayat.gujarat.gov.in .

[ref73] Parker JM , Brown JL, Hobbs NT, Boisseau NP, Letitiya D, Douglas-Hamilton I, Wittemyer G (2022) Social support correlates with glucocorticoid concentrations in wild African elephant orphans. Commun Biol 5: 630. 10.1038/s42003-022-03574-8. 35835816 PMC9283395

[ref74] Patel V , Varma J, Nimbalkar S, Shah S, Phatak A (2020) Prevalence and profile of bullying involvement among students of rural schools of Anand, Gujarat, India. Indian J Psychol Med 42: 268–273. 10.4103/IJPSYM.IJPSYM_172_19. 32612332 PMC7320736

[ref75] Pethiyagoda PDRS , Prasad GAT, Mahaulpatha WAD (2015) First report of a fatal accident of a saltwater crocodile (*Crocodylus Porosus*) due to electrocution in Sri Lanka. Wild Lanka 3: 139–143. http://dr.lib.sjp.ac.lk/handle/123456789/5998.

[ref76] Pineda-Galindo E , Cerda-Molina AL, Mayagoitia-Novales L, Matamoros-Trejo G, de la O C (2017) Biological validations of fecal glucocorticoid, testosterone, and progesterone metabolite measurements in captive stumptail macaques (*Macaca arctoides*). Int J Primatol 38: 985–1001. 10.1007/s10764-017-9992-7.

[ref77] Pooley S (2016) A cultural herpetology of Nile crocodiles in Africa. Conserv Soc 14: 391–405. 10.4103/0972-4923.197609. https://www.jstor.org/stable/26393261.

[ref78] Pooley S (2022) The challenge of compassion in predator conservation. Front Psychol 13: 977703. 10.3389/fpsyg.2022.977703. 36092072 PMC9454015

[ref79] Rice CJ , Sandman CA, Lenjavi MR, Baram TZ (2008) A novel mouse model for acute and long-lasting consequences of early life stress. Endocrinology 149: 4892–4900. 10.1210/en.2008-0633. 18566122 PMC2582918

[ref80] Romero LM (2002) Seasonal changes in plasma glucocorticoid concentrations in free-living vertebrates. Gen Comp Endocrinol 128: 1–24. 10.1016/S0016-6480(02)00064-3. 12270784

[ref81] Romero LM , Beattie UK (2022) Common myths of glucocorticoid function in ecology and conservation. J Exp Zool 337: 7–14. 10.1002/jez.2459. 33819389

[ref82] Romero LM , Wikelski M (2001) Corticosterone levels predict survival probabilities of Galapagos marine iguanas during El Nino events. PNAS 98: 7366–7370. 10.1073/pnas.131091498. 11416210 PMC34674

[ref83] Rudolph K , Fichtel C, Heistermann M, Kappeler PM (2020) Dynamics and determinants of glucocorticoid metabolite concentrations in wild Verreaux's sifakas. Horm Behav 124: 104760. 10.1016/j.yhbeh.2020.104760. 32330550

[ref84] Rusch TW , Angilletta MJ Jr (2017) Competition during thermoregulation altered the body temperatures and hormone levels of lizards. Funct Ecol 31: 1519–1528. 10.1111/1365-2435.12869.

[ref85] Schaebs FS , Wolf TE, Behringer V, Deschner T (2016) Fecal thyroid hormones allow for the noninvasive monitoring of energy intake in capuchin monkeys. J Endocrinol 231: 1–10. 10.1530/JOE-16-0152. 27460343

[ref86] Scheelings TF , Jessop TS (2011) Influence of capture method, habitat quality and individual traits on blood parameters of free-ranging lace monitors (*Varanus varius*). Aust Vet J 89: 360–365. 10.1111/j.1751-0813.2011.00815.x. 21864309

[ref87] Scheun J , Campbell R, Ganswindt A, McIntyre T (2021) Hot and bothered: alterations in faecal glucocorticoid metabolite concentrations of the sungazer lizard, *Smaug giganteus*, in response to an increase in environmental temperature. Afr Zool 56: 222–230. 10.1080/15627020.2021.1980103.

[ref88] Schoech SJ , Mumme RL, Moore MC (1991) Reproductive endocrinology and mechanisms of breeding inhibition in cooperatively breeding Florida scrub jays (*Aphelocoma c. coerulescens*). Condor 93: 354–364. 10.2307/1368951.

[ref89] Sideleau BM , Britton ARC (2013) An analysis of crocodilian attacks worldwide for the period of 2008-July 2013. In Proceedings of the 22nd working meeting of the IUCN-SSC crocodile specialist group, IUCN, Switzerland, pp. 110–113.

[ref90] Smith JE , Monclús R, Wantuck D, Florant GL, Blumstein DT (2012) Fecal glucocorticoid metabolites in wild yellow-bellied marmots: experimental validation, individual differences and ecological correlates. Gen Comp Endocrinol 178: 417–426. 10.1016/j.ygcen.2012.06.015. 22732084

[ref91] Somaweera R , Brien ML, Sonneman T, Didham RK, Webber BL (2019) Absence of evidence is not evidence of absence: knowledge shortfalls threaten the effective conservation of freshwater crocodiles. Glob Ecol Conserv 20: e00773. 10.1016/j.gecco.2019.e00773.

[ref92] Sumera S , Husna M, Laiba S, Aqsa C (2018) Changes in growth hormone and cortisol profile due to lead induced toxicity in Labeo rohita. TrJFAS 18: 921–926. 10.4194/1303-2712-v18_7_10.

[ref93] Swain S , Mishra SK, Pandey A, Kalura P (2022) Inclusion of groundwater and socio-economic factors for assessing comprehensive drought vulnerability over Narmada River basin, India: a geospatial approach. Appl Water Sci 12: 14. 10.1007/s13201-021-01529-8.

[ref94] Tavalieri YE , Alarcón R, Tschopp MV, Canesini G, Luque EH, Muñoz-de-Toro M, Galoppo GH (2021) Exposure to xenoestrogens alters the expression of key morphoregulatory proteins of oviduct adenogenesis in the broad-snouted caiman (*Caiman latirostris*). Aquat Toxicol 235: 105817. 10.1016/j.aquatox.2021.105817. 33853019

[ref95] Terwissen CV , Mastromonaco GF, Murray DL (2013) Influence of adrenocorticotrophin hormone challenge and external factors (age, sex, and body region) on hair cortisol concentration in Canada lynx (*Lynx canadensis*). Gen Comp Endocrinol 194: 162–167. 10.1016/j.ygcen.2013.09.010. 24080086

[ref96] Thirion F , Tellez M, Van Damme R, Bervoets L (2022) Trace element concentrations in caudal scutes from *Crocodylus moreletii* and *Crocodylus acutus* in Belize in relation to biological variables and land use. Ecotoxicol Environ Saf 231: 113164. 10.1016/j.ecoenv.2022.113164. 35007829

[ref97] Touma C , Palme R (2005) Measuring fecal glucocorticoid metabolites in mammals and birds: the importance of validation. Ann N Y Acad Sci 1046: 54–74. 10.1196/annals.1343.006. 16055843

[ref98] Uluwaduge P , Menike KE, Senevirathna EMTK, Pathirana GCL (2018) Mitigating the human-crocodile conflict in Sri Lanka: a study based on the Nilwala River area in Matara District. Procedia eng 212: 994–1001. 10.1016/j.proeng.2018.01.128.

[ref99] Utete B (2021) A review of the conservation status of the Nile crocodile (*Crocodylus niloticus* Laurenti, 1768) in aquatic systems of Zimbabwe. Glob Ecol Conserv 29: e01743. 10.1016/j.gecco.2021.e01743.

[ref100] Van Der Ploeg JAN , van Weerd M, Persoon GA (2011) A cultural history of crocodiles in the Philippines: towards a new peace pact? Environ Hist Camb 17: 229–264. 10.3197/096734011X12997574043008.

[ref101] Vashistha G , Deepika S, Dhakate PM, Khudsar FA, Kothamasi D (2020) The effectiveness of microsatellite DNA as a genetic tool in crocodilian conservation. Conserv Genet Resour 12: 733–744. 10.1007/s12686-020-01164-6.

[ref102] Voluntary Nature Conservancy (2017) 4th Charotar Crocodile Count- 2017. Voluntary Nature Conservancy, Gujarat, India.

[ref103] Voluntary Nature Conservancy (2018) 5th Charotar Crocodile Count- 2018. Voluntary Nature Conservancy, Gujarat, India, p. 30.

[ref104] Voluntary Nature Conservancy (2019) 6th Charotar Crocodile Count- 2019. Voluntary Nature Conservancy, Gujarat, India, p. 24.

[ref105] Voluntary Nature Conservancy (2020) 7th Charotar Crocodile Count- 2020. Voluntary Nature Conservancy, VallabhVidyanagar, Gujarat, Gujarat, India, p. 2.

[ref106] Voluntary Nature Conservancy (2021) 8th Charotar Crocodile Count- 2021. Voluntary Nature Conservancy, Gujarat, India, p. 12.

[ref107] Voluntary Nature Conservancy (2022) 9th Charotar Crocodile Count- 2022. Voluntary Nature Conservancy, Gujarat, India, p. 12.

[ref108] Vyas R (2013) Survey of vertebrate fauna along the select segments of some rivers of central Gujarat, India, In Ketan Tatu eds, *Jalaplavit* **4**: 80.

[ref109] Vyas R (2018) Results of the 2015 mugger crocodile (*Crocodylus palustris*) count at Vadodara, Gujarat, India. Reptiles Amphib 25: 20–25. 10.17161/randa.v25i1.14221.

[ref110] Vyas R (2023) The mysterious death of marsh crocodiles, *Crocodylus palustris*, Vadodara, Gujarat state, India. Reptiles Amphib 30: e18141–e18141. 10.17161/randa.v30i1.18141.

[ref111] Vyas R , Stevenson C (2017) Review and analysis of human and mugger crocodile conflict in Gujarat, India from 1960 to 2013. JoTT 9: 11016–11024. 10.11609/jott.3790.9.12.11016-11024.

[ref112] Vyas R , Vasava A (2019) Mugger crocodile (*Crocodylus palustris*) mortality due to roads and railways in Gujarat, India. Herpetol Conserv Biol 14: 615–626.

[ref113] Wallace KM , Leslie AJ, Coulson T (2011) Living with predators: a focus on the issues of human–crocodile conflict within the lower Zambezi valley. Wildl Res 38: 747–755. 10.1071/WR11083.

[ref114] Weingrill T , Gray DA, Barrett L, Henzi SP (2004) Fecal cortisol levels in free-ranging female chacma baboons: relationship to dominance, reproductive state and environmental factors. Horm Behav 45: 259–269. 10.1016/j.yhbeh.2003.12.004. 15053942

[ref115] Whitaker R , Whitaker Z (1984) Reproductive biology of the mugger (*Crocodylus palustris*). JBNHS 81: 291–317. https://biostor.org/reference/153657.

[ref116] Wingfield JC (2013) The comparative biology of environmental stress: behavioural endocrinology and variation in ability to cope with novel, changing environments. Anim Behav 85: 1127–1133. 10.1016/j.anbehav.2013.02.018.

[ref117] Yadav R , Yadav SM (2023) Evaluation of parametric postprocessing of ensemble precipitation forecasts of the NCMRWF for the Vishwamitri river basin. J Hydroinform 25: 349–368. 10.2166/hydro.2023.113.

